# Utility of a shortened Hasegawa Dementia Scale Revised questionnaire to rapidly screen and diagnose Alzheimer’s disease

**DOI:** 10.1002/agm2.12152

**Published:** 2021-06-08

**Authors:** Qian Gong, Masaki Ishii, Ouka Numata, Wenke Xie, Takeo Hirata

**Affiliations:** ^1^ Department of Medicine Okamotoishii Hospital Shizuoka Japan; ^2^ Department of Geriatric Medicine University of Tokyo Tokyo Japan; ^3^ Department of Medicine National Hospital Organization Tokyo Medical Center Tokyo Japan; ^4^ Chasuqi Cilao Clinic of Huhhot Huhhot China

**Keywords:** Alzheimer's disease, diagnostic ability, Hasegawa Dementia Scale Revised, region of interest, reliability, sensitivity, specificity, validity, voxel‐based specific regional analysis system for Alzheimer's disease

## Abstract

**Aims:**

The aim of this study was to analyze the sensitivity and specificity of a shortened Hasegawa Dementia Scale Revised (shortened HDS‐R) questionnaire and explore its utility for the rapid screening and diagnosis of Alzheimer’s disease (AD).

**Methods:**

We included 113 patients over the age of 60 years who visited our hospital from June 2018 to January 2021 including 70 subjects with AD and 43 healthy subjects. AD was diagnosed in accordance with the diagnostic criteria of the Diagnostic and Statistical Manual of Mental Disorders, 4th edition, and the standard HDS‐R questionnaire was used as a neuropsychological examination. The shortened HDS‐R questionnaire was composed of the first seven subdomains (1 to 7) of the HDS‐R questionnaire and excluded subdomains 8 and 9. Magnetic resonance imaging (MRI) was performed to calculate the degree of atrophy of the whole brain, hippocampus, and parahippocampal gyrus.

**Results:**

The cumulative contribution ratio of subdomains 1 to 7 of the HDS‐R questionnaire was as high as 94%, indicating that the construct validity of the shortened HDS‐R was very good. The correlation coefficient of the total scores of the shortened HDS‐R and the HDS‐R was 0.96, indicating that the criterion‐related validity was also very good. Furthermore, the shortened HDS‐R was significantly negatively correlated with the degree of atrophy in the whole brain, hippocampus, and parahippocampal gyrus, indicating that its concurrent validity was very good in relation to imaging parameters. Cronbach’s α coefficient of the shortened HDS‐R was 0.76, and the correlation coefficient of the item‐total correlation analysis was between 0.68 and 0.76, indicating that this questionnaire has high internal consistency and reliability. The total shortened HDS‐R score of the normal group (17.0 ± 1.9) was significantly higher than that of the AD group (8.6 ± 3.8), demonstrating that the total shortened HDS‐R score can be used to identify healthy individuals and patients with AD. When the cutoff score was 14 of 15, the sensitivity was 92.9% and the specificity was 88.4%. The diagnostic ability of the shortened HDS‐R was 91.2%, which indicates that it is similar to the full HDS‐R questionnaire as an AD screening tool.

**Conclusion:**

As a neuropsychological examination questionnaire for the screening and diagnosis of AD, the shortened HDS‐R had very high validity and reliability. Its sensitivity, specificity, and diagnostic ability were similar to those of the gold standard HDS‐R; therefore, it can be considered a concise and useful questionnaire for AD screening and diagnosis in the older population.

## Graphical abstract

The shortened HDS‐R had high validity and reliability. Its sensitivity, specificity, and diagnostic ability were similar to those of the gold standard HDS‐R; therefore, it can be considered a concise and useful questionnaire for AD screening and diagnosis.

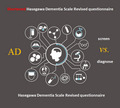

## 1 INTRODUCTION

The number of Americans living with Alzheimer's is growing fast. An estimated 6.2 million Americans age 65 and older are living with Alzheimer's dementia in 2021. Seventy‐two percent are age 75 or older according to Alzheimer's Association.^1^ In China, there are more than 10 million patients with dementia, of whom 6 million have AD, making China the first‐ranked country in the world in terms of its AD population. By 2050, the number of patients with AD in China is expected to reach 40 million, which will account for half of the global number of patients with AD.^2^ Statistics from the Ministry of Internal Affairs and Communications of Japan in 2016 revealed that the older population is increasing year by year, with one in three people over 65 years old.^3^ Furthermore, a survey report of the Japanese Ministry of Health, Labour, and Welfare predicted that the number of patients with dementia in Japan will be 7.3 million in 2025, with one out of five people over 65 years of age having dementia; they also predicted that this ratio may increase in the future.^4^

A diagnosis of dementia requires auxiliary examinations, including clinical symptom examinations, neuropsychological tests, and imaging examinations. The main international neuropsychological questionnaires include the Mini‐Mental State Examination (MMSE) and the Hasegawa Dementia Scale Revised (HDS‐R).^5^, ^6^ The MMSE includes 11 subdomains, as follows: (1) orientation to time; (2) orientation to place; (3) registration; (4) calculation; (5) delayed recall; (6) naming; (7) repeating; (8) three‐stage commands; (9) reading and obeying; (10) writing; and (11) coping.^5^ In contrast, the HDS‐R includes nine subdomains, as follows: (1) age; (2) time orientation; (3) space orientation; (4) immediate recall (registration); (5) calculation; (6) digits in reverse; (7) delayed recall; (8) item memory; and (9) speech fluency.^6^ The contents of the two are similar except for the delayed recall subdomain, and they are often used simultaneously as questionnaires for testing.^7^ Well‐trained medical staff should be responsible for testing with these questionnaires. The full score for each of these questionnaires is 30 points, and a higher score reflects better cognitive ability. Many studies have reported that the sensitivity of HDS‐R (93%) is higher than that of MMSE (82.8%),^6^, ^8^, ^9^ making the HDS‐R more suitable for screening for AD. In addition, previous clinical studies conducted on patients with AD and healthy controls have demonstrated that the diagnostic ability of MMSE is 90.2%, whereas the diagnostic ability of HDS‐R is 95.2%. Thus, the HDS‐R has a better diagnostic ability and facilitates the identification of patients with AD from healthy controls.^10^, ^11^ It takes an average of 6 to 10 minutes to implement the HDS‐R questionnaire.^12^ There is thus an urgent need for a more concise and effective questionnaire for both large‐scale screening and busy outpatient services. In recent work, we found a very high correlation between scores in the HDS‐R questionnaire as a whole and the same questionnaire after the deletion of subdomains 8 and 9. In the HDS‐R questionnaire, the eighth subdomain of memory requires a set of tools, such as a watch, pencil, spoon, key, and toothbrush, whereas the ninth subdomain of speech fluency requires patients to list as many vegetable names as possible, which are written down by the examiner. Together, the two subdomains take 3 to 5 minutes to complete. In addition, the set of tools required for subdomain 8 makes HDS‐R unsuitable for the screening of large‐scale populations, whereas subdomain 9 takes a relatively long time and has sex differences. We therefore removed these two subdomains from the questionnaire, which we called the shortened HDS‐R questionnaire. If the shortened HDS‐R questionnaire is a suitable substitute for the HDS‐R questionnaire, as we expect, it will provide a shorter and more feasible test tool for large‐scale screening. It will also be helpful in busy outpatient services by reducing the burden on patients as well as the workload of medical staff. In the present study, we therefore investigated the reliability and validity of the shortened HDS‐R questionnaire and conducted an exploratory study on the utility of the shortened HDS‐R by evaluating its sensitivity, specificity, and diagnostic ability.

## 2 METHODS

### 2.1 Study population

The study population consisted of the patients in our hospital from June 2018 to January 2021. AD was diagnosed by a senior clinician using the diagnostic criteria of the Diagnostic and Statistical Manual of Mental Disorders, 4th edition.^13^

Exclusion criteria included patients aged under 60 years, patients with other types of dementia, patients with severe liver and kidney disorders, and patients with cancer. A total of 113 patients were recruited for the study.

### 2.2 Shortened HDS‐R

The shortened HDS‐R contained the first seven items of the HDS‐R and had a maximum score of 20 points. The number of points per item were as follows: 1, 4, 2, 3, 2, 2, and 6 points for (1) age, (2) time orientation, (3) space orientation, (4) immediate recall, (5) calculation, (6) digits in reverse, and (7) delayed recall, respectively.

Brain magnetic resonance imaging (MRI) was performed to measure the degree of decreased brain volume in the medial temporal lobe.^14^, ^15^, ^16^, ^17^ Using the voxel‐based specific regional analysis system for Alzheimer’s disease (VSRAD), the volumes of interest (VOIs) of the hippocampus, parahippocampal gyrus, entorhinal cortex, and the whole brain were quantitatively evaluated. The measured results of the severity of VOI atrophy, extent of VOI atrophy, extent of gray matter (GM) atrophy, and ratio of VOI/GM atrophy were then calculated.^18^, ^19^, ^20^

As a new questionnaire, we first needed to evaluate the validity and reliability of the shortened HDS‐R. To measure the validity, an exploratory factor analysis was performed to calculate the cumulative contribution ratio, to evaluate the construct validity. Taking HDS‐R as the gold standard, the Pearson correlation coefficient with shortened HDS‐R was calculated to evaluate the criterion‐related validity. In addition, by calculating the Pearson correlation coefficient between the shortened HDS‐R and the severity of VOI atrophy, extent of VOI atrophy, extent of GM atrophy, and ratio of VOI/GM atrophy from the VSRAD, the concurrent validity was evaluated. To measure the reliability, Cronbach’s α coefficient was calculated, and an item‐total correlation analysis was conducted to evaluate its internal consistency.

To evaluate the utility of the shortened HDS‐R, the total shortened HDS‐R scores of the healthy group and the AD group were calculated, as were the means and standard deviations of the scores of each subdomain. The cutoff value was inferred to calculate the sensitivity, specificity, and diagnostic ability of the shortened HDS‐R.

### 2.3 Statistical analysis

For the qualitative data of the demographic characteristics, the Mantel–Haenszel or chi‐squared tests were used for comparisons between the two groups. For quantitative data of age, HDS‐R scores, and score of VSRAD, the independent *t* test or F test of the generalized linear model statistical analysis were performed between groups. Pearson correlation analysis was used to analyze the association between the HDS‐R or shortened HDS‐R scores and VSRAD scores. Data are expressed as the mean ± standard deviation for numerical variables, or as the number (%) for categorical variables. All hypothesis testing was two‐sided and *P* < 0.05 was taken as statistically significant. All analyses were performed using SAS version 9.3 (SAS Institute).

### 2.4 Informed consent

All patients who voluntarily participated in the study and their families received an explanation of the study summary and the protection of personal information, and informed consent was obtained. Ethics approval was obtained from the ethics committee of Okamoto Ishii Hospital, Shizuoka Prefecture.

## 3 RESULTS

### 3.1 Demographic results

In this study, 113 patients were included, including 43 men and 70 women. The mean age was 80.8 ± 6.7 years (range = 63‐98 years). There were 44 cases of hypertension, 14 cases of diabetes, 18 cases of hyperlipidemia, and six cases of smoking. There were 70 individuals in the AD group, and 43 individuals in the healthy group. The demographic results are shown in Table 1. The mean age of the patients in the AD group was 82.4 ± 6.4 years, and that of the healthy group was 78.2 ± 6.3 years; this difference was significant between the two groups (*P* = 0.001). However, there was no significant difference in the sex ratio between the two groups. In addition, there were no significant differences in the rates of hypertension, diabetes, hyperlipidemia, smoking, or other lifestyle diseases between the two groups (see Table 1).

**TABLE 1:** Demographic data

	AD group N = 70	Control group N = 43	*P* values
Age	82.4 ± 6.4	78.2 ± 6.3	0.001
Gender (male/female)	28/42	15/28	0.587
Complications			
Hypertension	30	14	0.276
Diabetes	9	5	0.845
Hyperlipidemia	13	5	0.327
Smoking	4	2	1.000

Abbreviation: AD, Alzheimer’s disease.

### 3.2 Validity of the shortened HDS‐R questionnaire

#### 3.2.1 Evaluation of construct validity

An exploratory factor analysis of HDS‐R revealed that the cumulative contribution ratio of the first seven subdomains of HDS‐R was 94%.

#### 3.2.2 Evaluation of criterion‐related validity

HDS‐R was used as the gold standard. A correlation analysis between the total shortened HDS‐R score and the total HDS‐R score was performed. The Pearson correlation coefficient revealed a significant correlation between the total scores of the two questionnaires (*r* = 0.95, *P* < 0.001).

#### 3.2.3 Evaluation of concurrent validity

The evaluation of coexistence validity was performed by calculating the correlations between cognitive dysfunction evaluations and imaging evaluations. The Pearson correlation coefficients between total shortened HDS‐R scores and the severity of VOI atrophy, extent of VOI atrophy, extent of GM atrophy, and ratio of VOI/GM atrophy were −0.49 (*P* < 0.001), −0.47 (*P* < 0.001), −0.38 (*P* < 0.001), and −0.33 (*P* < 0.004), respectively.

### 3.3 Reliability of the shortened HDS‐R questionnaire

The shortened HDS‐R questionnaire consisted of seven subdomains of the HDS‐R, with a maximum total score of 20 points. The means and standard deviations of each subdomain of the shortened HDS‐R are shown in Table 2. The mean score of the shortened HDS‐R was 11.8 ± 5.6 points (range = 0‐20 points).

**TABLE 2:** Analysis of the subdomains of the shortened HDS‐R (n = 113)

	Range	Mean	SD	I‐T correlation coefficient
1. Age	0‐1	0.8	0.4	0.64
2. Time orientation	0‐4	1.9	1.6	0.81
3. Space orientation	0‐2	1,4	0.7	0.72
4. Immediate recall	0‐3	2.7	0.8	0.53
5. Calculation	0‐2	1.2	0.8	0.65
6. Digital reverse singing	0‐2	1.0	0.8	0.67
7. Delayed recall	0‐6	2.7	2.2	0.85
Shortened HDS‐R	0‐20	11.8	5.6	
HDS‐R	0‐30	17.4	8.2	

Abbreviations: HDS‐R, Hasegawa Dementia Scale Revised; I‐T, item‐time; SD, standard deviation.

#### 3.3.1 Item‐total correlation analysis

The correlation analysis results among the seven subdomain scores and the total score are shown in Table 2. The highest Spearman’s rank correlation coefficient was that of delayed recall, which was 0.86 (*P* < 0.001). The lowest Spearman’s rank correlation coefficient, for immediate recall, was 0.53. The item‐total correlation coefficients of all subdomains were greater than 0.5 (*P* < 0.001).

#### 3.3.2 Cronbach’s α coefficient

Taking all 113 columns as the subjects, Cronbach’s α coefficient of the shortened HDDS‐R questionnaire was calculated, giving a result of 0.76. The Cronbach’s α coefficient of each subdomain was removed when calculating, and the coefficient range was within the range of 0.68 to 0.76, indicating no bias among the items.

### 3.4 Utility of the shortened HDS‐R questionnaire

#### 3.4.1 Differences in total shortened HDS‐R scores between the two groups

The mean total shortened HDS‐R score of the healthy group was 17.0 ± 1.9, which was significantly higher than that of the AD group, of 8.6 ± 3.8 (*P* < 0.001). The difference in total shortened HDS‐R scores between the AD and normal groups was 8.4 ± 3.8 points. After adjusting for age, the least squares means of the total HDS‐R scores for the AD and normal groups were 8.8 and 16.2 points, respectively (*P* < 0.000) This finding indicates that when the total shortened HDS‐R score was high, the possibility of the subject being from the healthy group was high. In contrast, when the total score was low, the possibility of AD was high.

#### 3.4.2 The sensitivity, specificity, and diagnostic ability of the shortened HDS‐R questionnaire

When the cutoff value for the shortened HDS‐R score was 14 of 20, the diagnostic ability of this questionnaire was 91.2%, its sensitivity was 92.9%, and its specificity was 88.4%.

## 4 DISCUSSION

The shortened HDS‐R questionnaire is composed of subdomains 1 to 7 of the HDS‐R and has a maximum total score of 20 points. The test takes only 3 to 5 minutes to perform and takes much less time to complete than the full HDS‐R questionnaire.

### 4.1 Validity of the shortened HDS‐R

Regarding the construct validity, the accumulative incidence rate of the first seven items of the HDS‐R was 94%; that is, the content validity of the shortened HDS‐R is good and can substitute functions of the HDS‐R in terms of content composition.

Regarding criterion‐related validity, HDS‐R has been demonstrated to have high reliability and validity, and has been used as the gold standard for screening and diagnosing dementia. The correlation coefficient between the shortened HDS‐R and the HDS‐R was as high as 0.96, with a high degree of correlation. It can therefore be considered that the shortened HDS‐R has good criterion‐related validity.

Regarding concurrent validity, pathological studies have shown that in the early stages of AD, atrophy occurs in the parahippocampal gyrus of the temporal lobe, and gradually expands to the cerebral cortex. In the present study, there were moderately negative correlations between shortened HDS‐R scores and the severity of VOI atrophy, extent of VOI atrophy, and extent of GM atrophy. This finding indicates that a lower score in the shortened HDS‐R is associated with more severe atrophy in the parahippocampal gyrus of the temporal lobe. Thus, the total shortened HDS‐R score may be used to infer the degree of atrophy of the parahippocampal gyrus. The results of the current study are consistent with those of previous HDS‐R studies on brain atrophy.^11^, ^21^, ^22^ From an imaging point of view, the concurrent validity is therefore very good.

### 4.2 Reliability of the shortened HDS‐R

All of the correlation coefficients in the item‐total correlation analysis were greater than 0.5; a positive correlation indicates high internal consistency. Moreover, Cronbach’s α coefficient was 0.76. According to the literature, a correlation coefficient of between 0.7 and 0.8 indicates high reliability.^10^ Thus, the shortened HDS‐R meets the reliability benchmark and has good internal consistency. We can therefore conclude that the shortened HDS‐R, which only uses the first seven subdomains of the HDS‐R, has satisfactory reliability.

### 4.3 Utility of the shortened HDS‐R

The difference in mean values of the shortened HDS‐R scores between the two groups was significant, and scores in the normal group were significantly higher than those in the AD group. A comparison of the sensitivity, specificity, false‐negative rate, false‐positive rate, and diagnostic ability between the shortened HDS‐R and the HDS‐R is shown in Table 3. A previous study reported that the sensitivity of HDS‐R is 91.7%, its specificity is 81.8%, and its diagnostic ability is 86%. ^11^ The sensitivity of HDS‐R in this study was 100.0%, its specificity was 82.7%, and its diagnostic ability was 92.0%. Furthermore, the sensitivity of the shortened HDS‐R was 92.9%, its specificity was 88.4%, and its diagnostic ability was 91.2%. Therefore, as an AD screening scale, the shortened HDS‐R has a similar performance to that of the HDS‐R questionnaire.

**TABLE 3:** Comparison of the utility of the shortened HDS‐R and the HDS‐R

	Shortened HDS‐R	HDS‐R
Cutoff score	14/15	20/21
Sensitivity (%)	92.9	100.0
Specificity (%)	88.4	82.7
False negative rate (%)	7.1	0.0
False positive rate (%)	11.6	17.3
Diagnostic ability (%)	91.2	92.0

Abbreviation: HDS‐R, Hasegawa Dementia Scale Revised.

### 4.4 Characteristics of the shortened HDS‐R

The shortened HDS‐R test took only 3 to 5 minutes to complete, which is approximately half the time of the HDS‐R test. This shortened timeframe reduces the burden on patients, and also reduces the workload of nurses/psychological technicians in busy clinics. The shortened HDS‐R omits the HDS‐R subdomain of memory, meaning that it does not require a set of tools, such as watches, pencils, keys, toothbrushes, and spoons. This feature makes the shortened HDS‐R questionnaire more suitable for large‐scale population screening work. With increases in the aged population and in patients with AD, the shortened HDS‐R is advantageous in that it provides a simple, quick, and effective test for AD. In addition, the shortened HDS‐R omits the HDS‐R subdomain of speech fluency. The subdomain requires subjects to list the names of vegetables. The testers write down the name of each vegetable, which takes a relatively long time (about 2‐3 minutes). In addition, most men who do not cook obtain low scores, which often causes a difference in scores between men and women. Thus, the deletion of this subdomain resolves the problem of sex differences in the HDS‐R. It also saves time, and may allow for computer‐based testing and on‐line diagnosis and treatment in the future.

### 4.5 Limitations of this study

All subjects were from the same hospital, and the sample size is small. In the future, it will be necessary to increase the number of subjects, to further explore the validity and utility of the shortened HDS‐R with a larger sample size.

## 5 CONCLUSIONS

The results of the present study indicate that the shortened HDS‐R is similar to the HDS‐R in terms of reliability and validity, and suggest that it avoids many of the shortcomings of the HDS‐R. The shortened version of the questionnaire takes 3 to 5 minutes only, meaning that it takes less time to test subjects compared with the full HDS‐R. The shortened HDS‐R may therefore be more suitable for rapidly and efficiently screening AD in older individuals, thus providing a more feasible test questionnaire for the screening and diagnosis of AD in the future.

## CONFLICTS OF INTEREST

There are no conflicts of interest to be reported by the authors of this study.

## AUTHOR CONTRIBUTIONS

*Writing of the manuscript:* Qian Gong, Masaki Ishii, Ouka Numata, Wenke Xie, and Takeo Hirata. *Study design:* Qian Gong and Masaki Ishii. *Literature review:* Qian Gong, Masaki Ishii, and Ouka Numata. *Data collection:* Qian Gong and Takeo Hirata. *Data coordination:* Qian Gong. *Data management:* Ouka Numata. *Statistical analysis:* Wenke Xie.
